# Long COVID and possible preventive options

**DOI:** 10.1007/s10787-023-01204-1

**Published:** 2023-06-21

**Authors:** Szilvia Sebők, Klara Gyires

**Affiliations:** 1 University Pharmacy Department of Pharmacy Administration, Faculty of Pharmaceutical Sciences, Semmelweis University, Hőgyes Endre U. 7-9., Budapest, 1092 Hungary; 2 Department of Pharmacology and Pharmacotherapy, Faculty of Medicine, Semmelweis University, Nagyvárad tér 4., Budapest, 1089 Hungary

**Keywords:** Long COVID, Risk factors, Potential managements, Clinical trials

## Abstract

Most of the people who suffered from COVID-19 fully recovered, but approximately 10–20% of them developed a wide variety of symptoms after they recover from their initial illness. Long COVID can develop at any patient; however, several studies suggest that the development of Long Covid syndrome may be linked to severity of acute illness. Some of the risk factors are hospitalization (with mechanical ventilation), Intensive Care Unit admission, age (over 50 years), gender (female) and comorbidities. Since the precise mechanism of Long COVID has not been clarified, neither the management of Long COVID-19 syndrome has been solved yet. Promising results have been published with vaccines as they effectively reduced the risk of Long COVID; however, other data suggest that vaccination results only partial protection in the post-acute phase of the disease. Recently, the orally effective antiviral agents (Paxlovid, molnupiravir) are preferred for outpatient management, and they highly reduce the progression of mild-to-moderate COVID-19 to severe one, and consequently, might reduce the development of Long COVID. Finally, recently, several clinical trials are in progress with either dietary supplements or drugs with different mechanisms of action. Additional information on the precise mechanisms, risk factors of Long COVID may result in successful preventive and therapeutic management of Long Covid 19 syndrome.

## Introduction

Long COVID or post-COVID refers to the prolonged symptoms which may be new or returning ones, that patients may develop after recovery from the initial, acute SARS-CoV-2 infection. The post-COVID symptoms are highly heterogeneous and complex, fatigue, dyspnea, sleep disorders, gastrointestinal disturbance, chronic cough, anxiety, depression, memory loss, concentration problems and a significant impairment in their quality of life have been reported; fatigue and neurocognitive complaints are the predominate ones. The symptoms can be mild to severe, may persist for months, and can fluctuate or relapses.

The Long Covid terminology is generally used for the description of the condition when the symptoms last far longer than it would be expected after recovery from SARS-CoV-2 infection. However, since both the course, the symptoms, the severity and the duration of Long COVID show great diversity, there is no consensus how to define it.

The lack of consensus is reflected by the numerous names that have been used for Long Covid syndrome, including: Post-COVID, chronic COVID syndrome, Long-hauler COVID-19, Long-haul COVID, Long-tail COVID, late sequelae of SARS-CoV-2 infection, post-acute sequelae of SARS CoV-2 infection and post-acute COVId-19 (Yong 2021).

## Definitions of Long COVID

Fernández-de-las-Peñas et al. (2021a, b) distinguished four stages in the course of Long COVID: a./4–5 weeks after the initial phase of the disease characterized by *infection-related symptoms,* b./from week 5 to 12 after the onset *acute post-COVID symptoms*, c./from week 12–24 after the initial period *Long post-COVID symptom,* and d./over 24 weeks after the acute infection *the persistent post-COVID symptoms (*Fernández-de-Las-Peñas et al. 2021a).

Another definitions by the same group distinguished two phases: *acute post-COVID* (from week 5 to week 12) and *chronic post-COVID* (lasting more than 12 weeks after symptom’s onset) (Fernández-de-las-Peñas 2022). Similarly, two phases, *the ongoing symptomatic* COVID-19 (from 4 to 12 weeks) and *the post-COVID-19 syndrome* (12 weeks or more) were suggested by Datta et al. (2020). Furthermore, according to Yong (2021) when the symptoms persist for more than 3 months after the onset of the disease, it can be taken as Long COVID.

The National Institute for Health and Care Excellence guideline (United Kingdom) distinguishes Long- and post-COVID conditions: *Long COVID* means the ongoing symptomatic COVID-19, where symptoms last for 4 to 12 weeks and *post-COVID-19* syndrome, where the symptoms persist beyond 12 weeks in the absence of an alternative diagnosis (NICE 2020).

Similarly, the World Health Organization (WHO) defines Long COVID as symptoms that persist for 3 months after the onset of COVID *infection* and the symptoms cannot be explained by an alternative diagnosis (Soriano et al. 2022)*.*

### Prevalence of Long-COVID syndrome after the acute SARS-CoV-2 infection

People infected with SARS-CoV-2 approximately 10–20% developed a wide variety of symptoms after they recover from their initial illness (Ballering et al. 2022); others have experienced higher prevalence, 30% of the development of Long COVID-19 in people infected with SARS-CoV-2 (Yoo et al. 2022). On the other hand, much lower (around 7%) prevalence of Post-Acute Sequelae of SARS-CoV-2 infection (PASC) was found by Xie et al. (2021), though it was consistently higher in people with poorer baseline health and in those who had more severe acute infection.

### Susceptibility to Long COVID

Long COVID can develop at any patient, also after mild-to-moderate cases and also in youngers, who were not admitted to hospital or even had asymptomatic COVID-19 (Dennis et al. 2021; Shah et al. 2021, Yong 2021). In addition, it has also been raised that neither the severity of the infection nor the intensity of the treatment the patients received, have any role in the development of Long COVID-19 (Crook et al. 2021).

In contrast, several studies suggest that the development of Long Covid symptoms may be linked to symptomatic COVID-19 infection; severity of illness, hospitalization (with mechanical ventilation), as well as sex (women) may have role in higher incidence of Long COVID (Baratta et al. 2021; Fernández-de-Las-Peñas et al. 2021b).

Others also confirmed and raised additional risk factors, for example the severity of illness (need for hospital or Intensive Care Unit /ICU/ admission), ventilatory support, age (over 50 years), gender (female) and comorbidities (asthma or previous respiratory disease, obesity and increased body mass index). On the other hand, several diseases, such as diabetes, hypertension, cancer and immunosuppression are risk factors for severity and mortality in the acute phase of COVID-19 infection, but no association has been proven with the development of post-COVID syndrome (Carod-Artal 2021). In consistence with the previous data, it has been found that patients who had to be admitted to hospital to the Intensive Care Unit (ICU) developed more frequently Long COVID, e.g., the prevalence of different post-COVID-19 symptoms were higher in patients who were treated in intensive care unit compared to ward patients, such as fatigue (72% vs. 60.3%), dyspnea (65.6% vs. 42.6%), attention and memory disorders (52% vs. 33.8%), and swallowing and language disorders (68.7% vs. 42.6%) were the most frequent symptoms (Halpin et al. 2021). Furthermore, patients (*n* = 119) treated in ICU, after recovery from the acute disease, 56% more likely to develop a neuropsychiatric disorder compared to non-ICU survivors (Taquet et al. 2021). Similarly, in patients who had been admitted to hospital for severe pneumonia 2 months after discharge, persistent symptoms were common, e.g., 68% had persistent fatigue, 57% sleep disorders, 39% persistent dyspnea or cough (D’Cruz et al. 2021).

In another study of 143 hospitalized patients with COVID-19, 87% of the patients had symptoms 2 months after being discharged and 40% reported a decrease in their quality of life, and fatigue persisted in over 50% for more than 60 days after the onset of symptoms (Carfì et al. 2020). Similar results have been published by Tomasoni et al (2021): patients with pneumonia due to SARS-CoV-2 infection complained about persistent physical symptoms (52%), anxiety (29%) and cognitive disorders (17%) 1–3 months after discharge (Tomasoni et al. 2021). Moreover, the health status of COVID-19 patients was evaluated at 6 and 12 months after hospital discharge. It was found that patients who needed respiratory support compared to patients who did not require, had higher probability of having symptoms at 6 and even in at 12 months. Consequently, it may be concluded that hospitalized COVID-19 patients with more severe symptoms in the acute phase may have higher risk of developing Long Covid symptoms (Lopez-Leon et al. 2021).

### Potential preventive options of Long COVID

#### Vaccines

The effect of vaccines on the development of Long COVID is inconsistent in the light of literature. The third dose of BNT162b2 (Pfizer–BioNTech) COVID-19 vaccine has important potential benefits; beyond reducing the illness, may prevent the COVID-19 sequelae after breakthrough infections. It was found that two or three doses of vaccine reduced the odds of developing Long COVID by 75 and 85%, respectively (Labos 2022; Ledford 2021). Accordingly, others have found that two doses of a COVID-19 vaccine resulted in 50% reduction of the risk of Long COVID. The data were obtained from self-reported information of 1.2 million UK individuals (Antonelli et al. 2022).

In contrast, though vaccination decreased the risk of death and post-acute sequelae, the protection was only partial in the post-acute phase of the disease (15%) according a study on US veterans (Al-Aly et al. 2022).

#### Antiviral compounds

As it was discussed above, several data suggest potential correlation between the severity of the acute infection of SARS**-**CoV-2 and the risk of the development of Long COVID-19. Therapeutic management of non-hospitalized (hospitalized) patients may prevent the progression of mild-to-moderate COVID-19 to severe one and, consequently, might reduce the risk of Long Covid.

Currently, for outpatient management, the orally effective antiviral agents are preferred such as nirmatrelvir plus ritonavir (Paxlovid) and molnupiravir. Recent findings suggest that oral treatment with nirmatrelvir plus ritonavir resulted in a 89% reduction of the risk of progression of mild-to-moderate COVID-19 to severe one (Hammond et al. 2022, Ledford 2022). Similarly, early treatment with molnupiravir (alternative therapy in non-hospitalized patients with mild-to-moderate COVID-19 who are at high risk of disease progression when Paxlovid is not available) reduced the risk of hospitalization (and death) in non-hospitalized, unvaccinated adults with mild-to-moderate COVID-19 who had at least one risk factor for severe COVID-19 illness (Jayk Bernal et al. 2022).

Parenteral preparations which also may prevent the progression of mild-to-moderate SARS-CoV-2 infection to severe one are the monoclonal antibody bebtelovimab as well as remdesivir, which is recommended both for the treatment of non-hospitalized and hospitalized patients, reduced the progression to mechanical ventilation for non-ventilated patients (Ali et al. 2022) (Table 1).

**Table 1:** Therapeutic management of non-hospitalized patients: antiviral agents and anti-SARS-CoV-2 monoclonal antibody

Oral	Parenteral
Paxlovid: *nirmatrelvir* (protease inhibitor, inhibition of the virus replication) + *ritonavi*r (slows the breakdown of nirmatrelvir by inhibiting CYP3A4)	Remdesivir: prodrug of an adenosine analogue, the active metabolite of remdesivir interferes with the action of viral RNA-dependent RNA polymerase and inhibits viral replication (Recommended for both non-hospitalized and hospitalized patients (WHO, September, 2022)
Molnupiravir: prodrug of N-hydroxycytidine (NHC). After phosphorylation in the cell to NHC triphosphate, it is incorporated into viral RNA and misdirects the viral polymerase and inhibits viral replication	Bebtelovimab: monoclonal antibody, isolated from a patient recovered from the COVID-19 disease, directed against the spike protein of the virus. (For the treatment of high-risk outpatients with mild-to-moderate COVID-19, if Paxlovid and remdesivir are not available.)

#### Glucocorticoids

COVID-19 is a multi-organ damage with substantial inflammatory component. In sever COVID-19, elevated levels of inflammatory markers have been observed. Though glucocorticoids are highly potent anti-inflammatory agents and show substantial benefits, their role in the therapy of COVID-19 has been debated. Recent study indicate that glucocorticoids (dexamethasone) have beneficial effect in hospitalized patients who are receiving respiratory support and the glucocorticoid therapy has been started after the first week, when already immunopathological elements dominate and the active viral replication (typical in the first week) have only a minor role. But no benefit (even a potential harm) was observed among patients who did not require respiratory support (oxygen or invasive mechanical ventilation) (The RECOVERY Collaborative Group 2021).

In contrast, it has not been clarified how glucocorticoids may influence the risk of the development of Long/post-COVID. Glucocorticoids as highly potent anti-inflammatory agent may reduce not only the mortality but also the progression of the disease (e.g., may prevent the need of invasive mechanical ventilation in patients who are given oxygen alone). As discussed above, there is a correlation between the severity of the initial infection of SARS-CoV-2 and the development of Long COVID-19 consequently, glucocorticoid treatment in acute phase of the disease might reduce the risk of the development of Long/post-COVID. Accordingly, a recent study showed that hospitalized COVID-19 patients, who received oral dexamethasone were less likely to experience persistent symptoms at 8-month follow-up (Milne et al. 2021). However, further studies are needed to reveal if glucocorticoid may reduce the risk of the development Long/post-COVID.

#### Ongoing and recently completed clinical trials with drugs and dietary supplements for the management of Long Covid syndrome

The precise mechanism of Long COVID has not been clarified. One of the potential mechanisms of Long COVID might be that lingering SARS-CoV-2 or fragments of the virus may further stimulate the immune system. Furthermore, it has also been raised that the infection induces production of antibodies that may damage the body’s own proteins, resulting in a cell/tissue damage for a longer period after the initial illness. It has also been observed that SARS-CoV-2 infection resulted in a formation of microscopic blood clots that reduce the oxygen supply of the tissues in a great extent (Ledford 2022).

Recently, several clinical trials are in progress in order to find effective, safe management of acute SARS-CoV-2 infection or Long Covid-19 syndrome. Most of the trials on Long COVID-19 analyzed the effect of either dietary supplements, e.g., mitoquinone (synthetic analogue of coenzyme Q10 with antioxidant property), vitamin K_2_, Vitamin D_3_, and N-acetyl-cysteine, or drugs, with different mechanism of action, for example, lithium, which has multiple mechanisms including anti-inflammatory actions, that may be relevant for treating Long COVID, naltrexone (opioid receptor antagonist, in lower doses has unique immune modulation activity), rintatolimod (stimulates the innate immune system by activating the TLR-3 receptors) amphetamine-dextroamphetamine (psychostimulants), vortioxetine (serotonin reuptake inhibitor, antidepressant), ipilimumab (monoclonal antibody, activates the immune system by targeting CTLA-4, a protein receptor that downregulates the immune system), pentoxifylline and ibudilast (both are phosphodiesterase inhibitors, pentoxifylline has also anti-inflammatory and immunomodulatory properties via adenosine A2A receptor), ivabradine (funny current (I_*f*_) inhibitor), and few antivirals, such as Paxlovid, remdesivir, favipiravir as well as vaccines (e.g., Pfizer/BioNTech BNT162b2 mRNA COVID-19 vaccine, Janssen COVID-19 Vaccine) (Table 2).

**Table 2:** Ongoing clinical trials on Long COVID **(**ClinicalTrials.gov website)*

	NCT number	Title	Interventions
1	NCT05618587	Effect of Lithium Therapy on Long COVID Symptoms	Drug: Lithium Drug: Placebo
2	NCT04997395	Feasibility of Cannabidiol for the Treatment of Long COVID	Drug: MediCabilis Cannabis sativa 50
3	NCT05595369	SARS CoV-2 Viral Persistence Study (PASC)—Study of Long COVID-19	Drug: Paxlovid Drug: Placebo
4	NCT05576662	Paxlovid for Treatment of Long Covid	Drug: Nirmatrelvir + Ritonavir Drug: Placebo + Ritonavir
5	NCT05220280	SOLIDARITY Finland Plus Long-COVID	Drug: Imatinib Drug: Infliximab
6	NCT05513560	Canadian Adaptive Platform Trial for Long COVID-19	Drug: Ibudilast Drug: Pentoxifylline Other: Placebo
7	NCT04978259	SOLIDARITY Finland Long-COVID (Remdesivir Long-term Follow-up Study of COVID Patients)	Drug: Remdesivir
8	NCT05592418	Study to Evaluate the Efficacy and Safety of Ampligen in Patients With Post-COVID Conditions	Drug: Rintatolimod Other: Placebo/Normal Saline
9	NCT04604704	Pilot Study Into LDN and NAD + for Treatment of Patients With Post-COVID-19 Syndrome	Drug: Naltrexone Dietary Supplement: NAD +
10	NCT05216614	Fluvoxamine to Augment Olfactory Recovery For Long COVID-19 Parosmia	Drug: Fluvoxamine Drug: Placebo
11	NCT04695704	Efficacy of Montelukast in Mild-moderate Respiratory Symptoms in Patients With Long-COVID-19:	Drug: Montelukast Other: placebo
12	NCT04948203	Assessing the Efficacy of Sirolimus in Patients With COVID-19 Pneumonia for Prevention of Post-COVID Fibrosis	Drug: Sirolimus
13	NCT05507372	Treatment for post acute COVID-19 Syndrome	Drug: Pimozide
14	NCT04904536	Statin TReatment for COVID-19 to Optimise NeuroloGical recovERy	Drug: Atorvastatin Other: Standard Care
15	NCT05497089	Temelimab as a Disease Modifying Therapy in Patients With Neuropsychiatric Symptoms in Post-COVID 19 or PASC Syndrome	Drug: Temelimab Drug: Placebo
16	NCT05350774	Immunotherapy for Neurological Post-Acute Sequelae of SARS-CoV-2	Drug: IV immunoglobulin Drug: IV normal saline Drug: IV methylprednisolone
17	NCT05430152	Low-dose Naltrexone for Post-COVID Fatigue Syndrome	Drug: Low-Dose Naltrexone Other: Placebo
18	NCT05096884	Post-Acute Sequelae of Coronavirus-19 (COVID-19) With Dyspnea on Exertion And Associated TaChycardia TrEatment Study	Drug: Metoprolol Succinate
19	NCT05047952	Vortioxetine for post-COVID-19 Condition	Drug: Vortioxetine Drug: Placebo
20	NCT05228899	Zofin to Treat COVID-19 Long Haulers	Drug: Zofin Other: Placebo
21	NCT05052307	A Real-world Evidence Study of BNT162b2 mRNA Covid-19 Vaccine in Brazil	Drug: Pfizer/BioNTech BNT162b2 mRNA COVID-19 vaccine, CoronaVac COVID-19 vaccine, ChAdOx1 nCoV-19 vid-19 Vaccine, Janssen COVID-19 Vaccine

*In the ClinicalTrials.gov database, on 29.11.2022, 333 hits were recived for the search term “long covid”. Among the ongoing clinical trials, drug intervention was performed in 21 cases. For scope reasons, studies using drugs and dietary supplements in the experimental stage have not been included in the table

The results of completed (by 24 November, 2022) clinical trials of drugs or dietary supplements on Long COVID syndrome are summarized on Table 3. From the 12 trials, more than half was ended as “no results”. The effect of dietary supplement Formula C was studied on the symptoms of Post-Acute COVID-19 Syndrome and improvement was experienced. However, limitation of the trial is the small number of participants (*n* = 32) and the lack of a true placebo. Leronlimab (humanized monoclonal antibody against the CCR5 receptor located on T lymphocytes) reduced several raw symptom scores in participants compared to control, but the mean symptom score changes for leronlimab vs placebo were statistically not significant. The clinical trial with Zilucoplan (complement C5 inhibitor) aimed to study the inhibition of post-COVID-19 acute lung injury, and the lung repair mechanisms. The results of the trial showed a relevant respiratory and clinical improvement in hypoxemic COVID-19 patients with systemic inflammation (Table 3).

**Table 3:** Clinical trials of drugs or dietary supplements on Long COVID completed by 24 November, 2022 (ClinicalTrials.gov database)*

	NCT number	Title	Aim	Interventions	Study design	Population	Results
1	NCT04871815	Effects of Sodium Pyruvate Nasal Spray in COVID-19 Long Haulers	To examine the effects of N115 (Sodium pyruvate nasal spray) treatment on the symptoms associated with COVID-19 Long Haulers	Drug: sodium pyruvate nasal spray	Allocation: N/A Intervention Model: Single Group Assignment Masking: None (Open Label) Primary Purpose: Treatment	Enrollment: 22 Age: 18 Years to 40 Years (Adult) Sex: All	Primary outcome data are available
2	NCT04795557	Efficacy of Adaptogens in Patients With Long COVID-19	To demonstrate possible efficacy of adjuvant treatment with ADAPT-232 in decreasing the duration of the convalescence, alleviation of fatigue, headache, attention deficit, difficult and rapid respiration, depression, anxiety and other symptoms of Long COVID-19 during rehabilitation period	Dietary Supplement: ADAPT-232 oral solution Other: Placebo oral solution	Allocation: Randomized Intervention Model: Parallel Assignment Masking: Quadruple (Participant, Care Provider, Investigator, Outcomes Assessor) Primary Purpose: Treatment	Enrollment: 100 Age: 18 Years and older (Adult, Older Adult Sex: All	No results
3	NCT04880161	A Study to Evaluate Ampion in Patients With Prolonged Respiratory Symptoms Due to COVID-19 (Long COVID)	To evaluate the safety of Ampion (low molecular weight filtrate of human serum albumin with the in vitro ability to modulate inflammatory cytokine levels) and the clinical outcomes in patients with Long COVID	Biological: Inhaled Ampion Other: Inhaled Placebo	Allocation: Randomized Intervention Model: Parallel Assignment Masking: Quadruple (Participant, Care Provider, Investigator, Outcomes Assessor) Primary Purpose: Treatment	Enrollment: 32 Age: 18 Years and older (Adult, Older Adult) Sex: All	Results Submitted, but is not yet publicly available
4	NCT05311852	Effects of PEA-LUT on Frontal Lobe Functions and GABAergic Transmission in Long-Covid Patients	To test the possible therapeutic effects of an 8-week therapy with PEA-LUT on GABAB-ergic neurotransmission, LTP-like synaptic plasticity, indexed with transient potentiation of motor evoked potentials (MEP) amplitude after repetitive TMS given as intermittent theta burst stimulation (iTBS) in Long COVID patients with cognitive complaints and fatigue	Dietary Supplement: palmitoylethanolami co-ultramicronized with antioxidant flavonoid luteolin (PEA-LUT) Dietary Supplement: Placebo	Allocation: Randomized Intervention Model: Parallel Assignment Masking: Double (Participant, Investigator) Primary Purpose: Treatment	Enrollment: 34 Age: 18 Years and older (Adult, Older Adult) Sex: All	No results
5	NCT04828668	Study to Evaluate Benefits and Safety of Endourage Formula C^™^ Oral Drops in People With Post-Acute COVID-19 Syndrome	To evaluate the clinical benefits of Formula C on symptoms in adults who have documented PACS or persistence of effects consistent with COVID-19 and to assess the impact of Formula C on quality-of-life (QOL) in persons with PACS. The secondary objective was to assess the safety of Formula C in persons with PACS	Dietary Supplement: Targeted Wellness Formula C^™^ Sublingual Drops- 1200 mg–30 mL (Formula C) Dietary Supplement: Placebo	Allocation: Randomized Intervention Model: Crossover Assignment Masking: Single (Participant) Primary Purpose: Other	Enrollment: 32 Age: 18 Years to 75 Years (Adult, Older Adult) Sex: All	Given that both groups demonstrated improvement, both formulations may be contributing to these findings. Limitations include the small number of participants, the lack of a true placebo, and limited time on study products (Young et al. 2022)
6	NCT05152849	Efficacy, Safety, Tolerability of AXA1125 in Fatigue After COVID-19 Infection	To compare the effects of AXA1125, an orally active mixture of amino acids, compared to placebo, on improving muscle function (metabolism) following moderate exercise in subjects with fatigue-Predominant Post-Acute Sequelae of SARS-CoV-2 as well as the safety and tolerability of AXA1125. Subjects will take one dose of AXA1125 or a placebo twice daily for 28 days	Drug: AXA1125 Drug: Placebo	Allocation: Randomized Intervention Model: Parallel Assignment Masking: Triple (Participant, Care Provider, Investigator) Primary Purpose: Treatment	Enrollment: 41 Age: 18 Years to 64 Years (Adult) Sex: All	No results
7	NCT04652518	LYT-100 in Post-acute COVID-19 Respiratory Disease	To evaluate the Safety and Efficacy of Deupirfenidone (LYT-100) in Post-acute COVID-19 Respiratory Disease	Drug: LYT-100 Other: Placebo	Allocation: Randomized Intervention Model: Parallel Assignment Masking: Triple (Participant, Investigator, Outcomes Assessor) Primary Purpose: Treatment	Enrollment: 185 Age: 18 Years to 80 Years (Adult, Older Adult) Sex: All	No results
8	NCT04960215	Coenzyme Q10 as Treatment for Long Term COVID-19	To investigate the effect of high-dose Coenzyme Q10 on number and severity of self-reported symptoms in patients with Long Term COVID-19	Drug: Coenzyme Q10 Drug: Placebo	Allocation: Randomized Intervention Model: Crossover Assignment Masking: Quadruple (Participant, Care Provider, Investigator, Outcomes Assessor) Primary Purpose: Treatment	Enrollment: 121 Age: 18 Years and older (Adult, Older Adult) Sex: All	No results
9	NCT04699097	The Effect of Azithromycin Use on Conduction System of Heart in COVID-19 Positive Children	To evaluate the effect of Azithromycin on ventricular repolarization in COVID-19 positive pediatric patients	Drug: Azithromycin	Observational Model: Case-Only Time Perspective: Prospective	Enrollment: 105 Age: 1 Year to 18 Years (Child, Adult) Sex: All	No results
10	NCT04814914	An observational clinical study to evaluate COVID-19 symptoms in “Long Hauler” patients who participated in K031-120 or K032-120	To evaluate the presence of symptoms of COVID-19 infection in patients who participated in K031-120 or K032-120 (“long hauler”)	Other: KB109 is a novel glycan + Self Supportive Care Other: Self Supportive Care (SSC) Alone	Observational Model: Cohort Time Perspective: Prospective	Enrollment: 333 Age: 18 Years and older (Adult, Older Adult) Sex: All	No results
11	NCT04382755	Zilucoplan^®^ in improving oxygenation and short- and long-term outcome of COVID-19 patients with acute hypoxic respiratory failure	The hypothesis of the proposed intervention is that Zilucoplan^®^ (complement C5 inhibitor) has profound effects on inhibiting acute lung injury post-COVID-19, and can promote lung repair mechanisms, that lead to a 25% improvement in lung oxygenation parameters. This hypothesis is based on experiments performed in mice showing that C5a blockade can prevent mortality and prevent ARDS in mice with post-viral acute lung injury	Drug: Zilucoplan^®^ Drug: Placebo	Allocation: Randomized Intervention Model: Parallel Assignment Masking: None (Open Label) Primary Purpose: Treatment	Enrollment: 81 Age: 18 Years and older (Adult, Older Adult) Sex: All	Complement C5 inhibition with zilucoplan led to numerically relevant respiratory and clinical improvements in hypoxemic COVID-19 patients with systemic inflammation, Zilucoplan lowered serum C5b-9 (*p* < 0.001) and interleukin-8 (*p* = 0.03) concentration compared with control. No relevant safety differences between the zilucoplan and control group were identified (De Leeuw et al. 2022)
12	NCT04678830	COVID-19 Long-Haulers Study	To assess the safety and efficacy of leronlimab (PRO 140) administered as weekly subcutaneous injections in subjects experiencing prolonged symptoms (> 12 weeks) of COVID-19	Drug: Placebos Drug: Leronlimab (700 mg)	Allocation: Randomized Intervention Model: Parallel Assignment Masking: Quadruple (Participant, Care Provider, Investigator, Outcomes Assessor) Primary Purpose: Treatment	Enrollment: 56 Age: 18 Years and older (Adult, Older Adult) Sex: All	The mean symptom score changes from baseline to the latest available time point from day 30–56 for leronlimab vs placebo were –16.0 and –12.0, respectively; adjusting for prespecified covariates, the adjusted mean difference was –1.0 (not statistically significant). For several symptoms, there was a numerically higher percentage of participants with reduced raw symptom scores for leronlimab compared with placebo treatment reaching borderline statistical significance without correction for multiple comparisons due to the exploratory nature of this pilot study (Gaylis et al. 2022)

*In the ClinicalTrials.gov database, 326 results were found for the keywords Long COVID, of which 58 trials studies were completed on 24.11.2022. Out of the completed trials, 15 involved the use of a drug or dietary supplements. 3 studies related to acute therapy, these were not relevant from the point of view of Long COVID

## Concluding remarks

Survivors of COVID-19 may have long-lasting symptoms. If Long COVID developed, its symptomology is very different and its management depends on which organ system has been damaged. Randomized trials are in progress to test drugs able to attenuate the different symptoms. The tested drugs have different mechanism of action, and several of them are repurposing drugs that already have approved for other conditions.

Though several mechanisms have been raised, the precise cause of Long COVID is unclear. Consequently, at present, no pharmacologic agent has been known that effectively reduces or abolishes the symptoms of Long COVID. One of the assumption has raised that the coronavirus after the acute infection is lingering in different tissues (e.g., in intestine, liver, and brain) where it causes damage. On the other hand, initial infection can induce production of antibodies and additional immunological reactions which can attack the body’s own tissues after the acute infection has already passed. Vaccination against SARS-CoV-2 considerably reduces the infection rates and the severity of symptoms as well as the development of Long COVID, though data on the protective role of vaccines are inconsistent. Therefore, further studies are needed to determine the effect of SARS-CoV-2 vaccination on Long COVID. Furthermore, since correlation between the severity of the acute infection of SARS-CoV-2 and the risk of the development of Long COVID-19 has been found, the orally effective as well as the parenteral antiviral agents that prevent the progression of mild-to-moderate COVID-19 to severe one might reduce also the risk of Long COVID. Finally, the role of different factors, such as gender, age, comorbidities, and severity of initial COVID-19 disease, should also be considered and clarified in the risk of post-COVID-19 condition.

## Data Availability

Not applicable.
